# Quantifying Intrinsic and Extrinsic Variability in Stochastic Gene Expression Models

**DOI:** 10.1371/journal.pone.0084301

**Published:** 2013-12-31

**Authors:** Abhyudai Singh, Mohammad Soltani

**Affiliations:** 1 Department of Electrical and Computer Engineering, University of Delaware, Newark, Delaware, United States of America; 2 Department of Biomedical Engineering, University of Delaware, Newark, Delaware, United States of America; 3 Department of Mathematical Sciences, University of Delaware, Newark, Delaware, United States of America; University of Erlangen-Nuremberg, Germany

## Abstract

Genetically identical cell populations exhibit considerable intercellular variation in the level of a given protein or mRNA. Both intrinsic and extrinsic sources of noise drive this variability in gene expression. More specifically, extrinsic noise is the expression variability that arises from cell-to-cell differences in cell-specific factors such as enzyme levels, cell size and cell cycle stage. In contrast, intrinsic noise is the expression variability that is not accounted for by extrinsic noise, and typically arises from the inherent stochastic nature of biochemical processes. Two-color reporter experiments are employed to decompose expression variability into its intrinsic and extrinsic noise components. Analytical formulas for intrinsic and extrinsic noise are derived for a class of stochastic gene expression models, where variations in cell-specific factors cause fluctuations in model parameters, in particular, transcription and/or translation rate fluctuations. Assuming mRNA production occurs in random bursts, transcription rate is represented by either the burst frequency (how often the bursts occur) or the burst size (number of mRNAs produced in each burst). Our analysis shows that fluctuations in the transcription burst frequency enhance extrinsic noise but do not affect the intrinsic noise. On the contrary, fluctuations in the transcription burst size or mRNA translation rate dramatically increase both intrinsic and extrinsic noise components. Interestingly, simultaneous fluctuations in transcription and translation rates arising from randomness in ATP abundance can decrease intrinsic noise measured in a two-color reporter assay. Finally, we discuss how these formulas can be combined with single-cell gene expression data from two-color reporter experiments for estimating model parameters.

## Introduction

Genetically identical cell populations exposed to same extracellular environment exhibit considerable variability in gene expression [1]–[5]. This variation in the level of a given protein is often referred to as *gene expression noise*. Increasing evidence suggests that noise plays important functional roles in many cellular processes. For example, tight control of expression noise is vital for optimal functioning of housekeeping proteins [6]–[8], and diverse diseased states have been attributed to an elevated expression noise [9]–[11]. Not surprisingly, genes actively use different regulatory mechanism to reduce stochastic fluctuations in protein levels [12]–[22], [22]–[25]. Expression noise is also exploited to drive genetically identical cells to different cell-fates [26]–[31], and to buffer cellular populations from hostile changes in the environment [27], [32]–[34].

Gene expression noise can be decomposed into intrinsic and extrinsic noise [35]–[37]. More specifically, *intrinsic noise* is the protein variability that arises from the inherent stochastic nature of biochemical reactions associated with transcription, translation, mRNA and protein degradation. Given that many mRNA species are present at low copy numbers inside cells, random birth and death of individual mRNA transcripts generates considerable intrinsic noise [38]–[41]. Let *Z* be any cell-specific factor (such as cell cycle stage, abundance of RNA polymerases/ribosomes, cellular environment, etc.) that affects expression of a given gene. Then, cell-to-cell differences in *Z* will create intercellular variability in gene expression, that is referred to as *extrinsic noise* Variations in *Z* induce fluctuations in model parameters (such as the transcription and translation rate), and extrinsic noise can be effectively quantified through analysis of deterministic gene expression models with corresponding parameter fluctuations [42].

**Figure 1:**
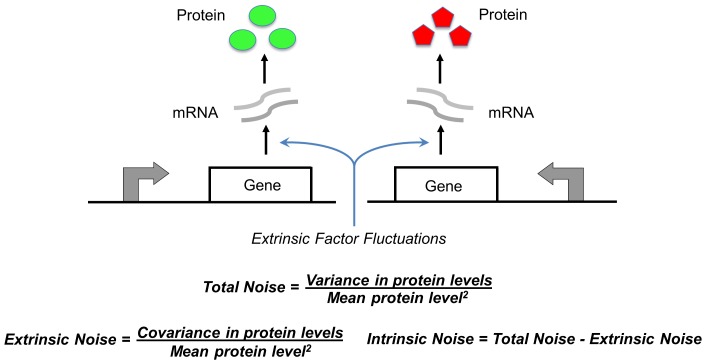
Decomposing gene expression variability into extrinsic and intrinsic noise using a two-color reporter assay. Two identical copies of a promoter express two different reporter proteins. Correlation in reporter levels is a measure of extrinsic noise that arises from cell-to-cell differences in shared cellular factors. Intrinsic noise is the protein variability that is not accounted for by extrinsic noise, and typically originates from the inherent stochastic nature of biochemical processes.

We define intrinsic and extrinsic noise in the context of a two-color experiment, where the gene of interest is duplicated inside the cell (Figure 1). Consider two identical copies of a promoter that express two different reporter proteins 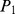 and 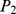. Let 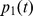 and 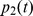 denote the level of these proteins at time 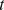 inside the cell. Since cell-specific factor 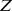 is common to both copies of the gene, cell-to-cell variations in 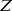 will make 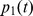 and 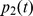 correlated. The contribution of 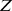 to expression noise is quantified via the extrinsic noise defined as
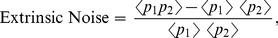
(1)and is related to the covariance between reporter levels. If reporter levels are perfectly correlated, and assuming 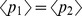, 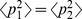,
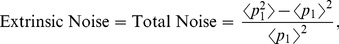
(2)which is the total noise in protein level measured by its coefficient of variation squared. Intrinsic noise is the protein variability that is not accounted for by extrinsic noise, and is defined as 
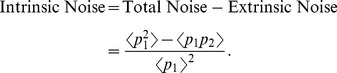
(3)


In summary, a two-color assay can be used to decompose the total protein noise level into intrinsic and extrinsic noise components, computed via (1) and (3), respectively.

Analytical formulas for intrinsic and extrinsic noise are derived for a class of stochastic gene expression models with fluctuations in the transcription or translation rate. Assuming mRNA production occurs in random bursts, transcription rate is represented by either the burst frequency (how often the bursts occur) or the burst size (number of mRNAs produced in each burst). Our results show that fluctuations in the transcription burst frequency enhance extrinsic noise but do not affect the intrinsic expression noise. However, fluctuations in the transcriptional burst size or mRNA translation rate increase both intrinsic and extrinsic noise. A recent study has implicated fluctuations in ATP levels as a major driver of gene expression variability [43]. Since ATP affects both transcription and translation, simultaneous fluctuations in multiple model parameters is investigated. Interestingly, simultaneous fluctuations in the transcription and translation rates decrease intrinsic noise in certain parameter regimes. Finally, usefulness of these formulas in interpreting two-color reporter experiments and estimating model parameters is discussed.

## Gene Expression with Constant Parameters

We begin by introducing the standard stochastic gene expression model [44]–[47], where all model parameters are fixed, and expression variability arises due to the stochastic nature of transcription and translation processes.

### Model Formulation

**Figure 2:**
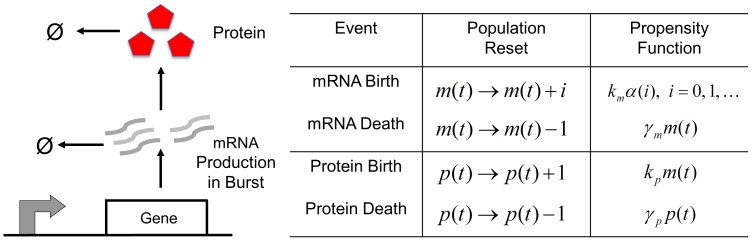
Model formulation. Schematic of the gene expression model (left). The stochastic model consists of four events that occur randomly at exponentially-distributed time intervals. Discrete changes in the mRNA (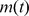) and protein (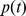) population count for different events are shown in the second column of the table. Third column lists the event propensity function that determines how often an event occurs.

Transcription has been shown to occurs in “bursts” with each burst producing multiple mRNA copies [48]–[53]. Assume mRNAs are produced in bursts of size 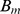 that occur at a rate 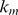. We refer to 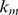 and 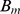 as the *transcriptional burst frequency and burst size*, respectively. Consistent with measurements [50], 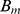 is assumed to be a geometrically distributed random variable with probability distribution
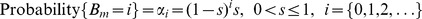
(4)and mean burst size 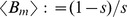. Proteins are produced from each mRNA at a translation rate 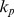. Finally, mRNAs and proteins degrade at constant rates 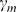 and 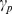, respectively. The stochastic model considers transcription, translation and degradation as probabilistic events that occur at exponentially-distributed time intervals [54], [55]. Moreover, whenever a particular event occurs, the mRNA and protein population count is reset accordingly. Let 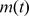 and 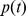 denote the number of molecules of the mRNA and protein at time 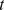, respectively. Then, the reset in 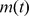 and 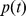 for different events is shown in the second column of the table in Figure 2. The third column lists the propensity functions 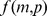 which determine how often an event occurs. In particular, the probability that a particular event will occur in the next infinitesimal time interval 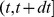 is given by 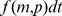.

### Computation of Intrinsic Noise

It is relatively straight forward to derive differential equations describing the time evolution of the different statistical moments of the mRNA and protein count. For the above model, the time-derivative of the expected value of any differentiable function 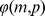 is given by
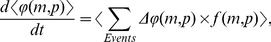
(5)where 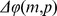 is the change in 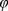 when an event occurs, 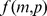 is the event propensity function, and 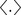 represents the expected value [56], [57]. Using the resets and propensity functions in Figure 2 this corresponds to
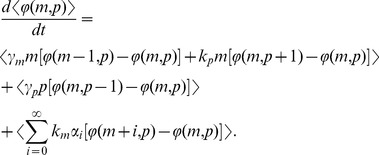
(6)


Choosing 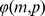 as 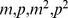 and 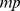 in the above equation yields
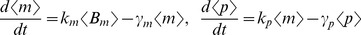
(7a)

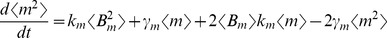
(7b)

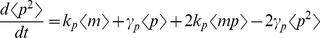
(7c)

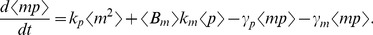
(7d)


Setting the left-hand-side of (7) to zero and solving for the moments results in the following steady-state mean protein and mRNA levels
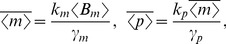
(8)where 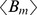 is the mean transcriptional burst size and 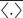 represents the steady-state expected value. As done in previous studies of intrinsic and extrinsic noise [35], [36], [58], the *steady-state coefficient of variation squared* (variance divided by mean squared) is used as a metric for quantifying the extent of variability/noise in protein copy numbers. From the steady-state protein variance and mean we obtain
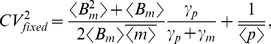
(9)which represents the total intrinsic noise in protein level for fixed parameters. As 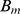 is geometrically distributed, 
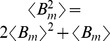
, and (9) reduces to



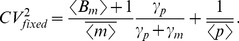
(10)The first term on the right-hand-side of (10) represents the noise in mRNA copy numbers that is transmitted to the protein level [15], [46]. The second term is the Poissonian noise arising from random birth-death of protein molecules. Next, the noise additional to (10) that comes from fluctuations in individual model parameters (such as 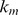, 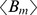 and 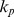) is quantified.

## Transcription Burst Frequency Fluctuations

Consider a cell-specific factor 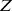 at the transcriptional level (such as a transcription factor). Then, fluctuations in 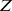 can either affect the transcriptional frequency 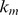 or burst size 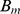 in the model. The former case of burst frequency fluctuations is considered first.

### Modeling Parameter Fluctuations

Let 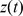 denote the level of a cellular factor 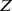 inside the cell at time 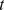. Fluctuations in 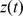 are modeled through a simple birth-death process with probabilities of formation and degradation in the infinitesimal time interval 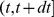 given by
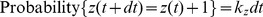
(11a)

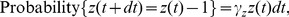
(11b)where 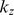 and 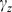 represent the production and degradation rate of 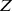, respectively. For the process described in (11), the steady-state mean, coefficient of variation squared 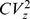 and the auto-correlation function 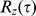 are given by



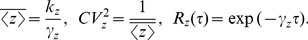
(12)Thus by changing 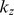 and 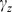, both the extent and time-scale of fluctuations in 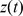 can be independently modulated. Note the inverse relationship between 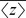 and 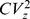 implies Poisson statistics. Fluctuations in 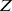 are incorporated in the model by assuming that the transcription burst frequency is no longer a constant but given by 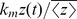, making it a random process with mean 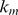 and coefficient of variation squared 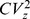. Throughout this manuscript, 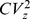 represents the extent of parameter fluctuations. Since 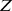 similarly affects expression of both copies of the gene in a two-color assay, fluctuations in 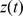 make reporter levels correlated in Figure 1 and induce extrinsic noise.

### Computation of Total Noise

The stochastic model consists of six birth-death events that change cellular factor, mRNA and protein copy numbers by integer amounts. Using the propensity functions in Figure 2 and (11) in (5) we obtain
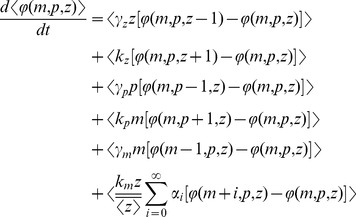
(13)for any differentiable function 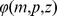. Appropriate choices of 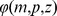 result in



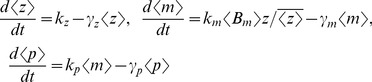
(14a)

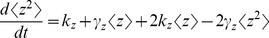
(14b)

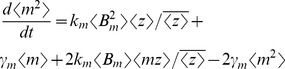
(14c)

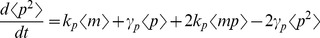
(14d)

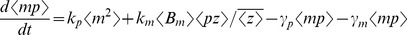
(14e)

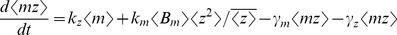
(14f)

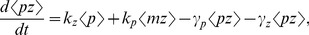
(14g)which yield the steady-state variability in protein level as



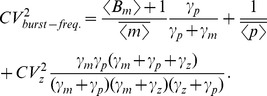
(15)The first two terms on the right-hand-side of (15) represent the noise level with fixed parameters (Eq. (10)). The third term is the additional noise due to burst frequency fluctuations. Next, (15) is decomposed into intrinsic and extrinsic noise components as measured by the two-color reporter assay (Figure 1).

### Computation of Intrinsic and Extrinsic Noise

Extrinsic noise can be approximated by the coefficient of variation squared of the protein level in a deterministic gene expression model with corresponding parameter fluctuations [42]. The deterministic counterpart to the stochastic model is the set of ordinary differential equations
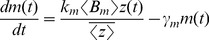
(16a)

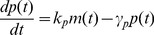
(16b)driven by the stochastic process 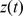 defined in (11). For this hybrid model, where some states are continuous and other are discrete, the time derivate of 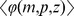 is given by (see Theorem 1 in [59])
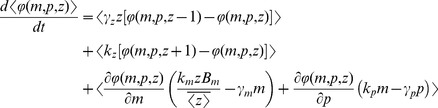
(17)and leads to moment dynamics identical to (14) except for



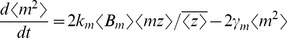
(18a)

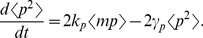
(17)


Quantification of protein noise level from (14) (with (14c)–(14d) replaced by (18a)–(18b)) gives the extrinsic noise, which is subtracted from (15) for the intrinsic noise. This analysis results in
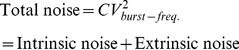
(19a)

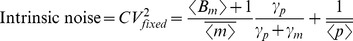
(19b)

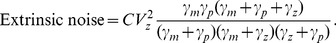
(19c)


As expected, extrinsic noise increases with extent of parameter fluctuations 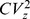. On the contrary, intrinsic noise is independent of 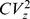 and is equal to 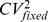. An important limit considered previously is the case where parameter values (in this case transcription burst frequency) are drawn from a static distribution [36]. In our model, this corresponds to a scenario where the time-scale of fluctuations in 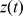 are slow compared to mRNA/protein turnover rates. When 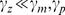, Eq. (19c) reduces to 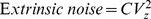, and this result is consistent with previous calculations of extrinsic noise for parameter values drawn from a static distribution (see Eq. 25 in [36]).

## Transcription Burst Size Fluctuations

Consider an alternative scenario of a fixed transcription burst frequency but varying burst size. Assume mRNAs are produced in geometrically distributed bursts with mean 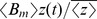, where 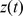 is the level of the cellular factor inside the cell at time 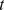. This implies
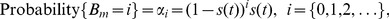
(20)and mean burst size


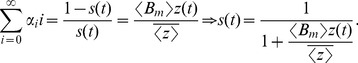
(21)

### Computation of Total Noise

Time derivative of statistical moments is obtained from
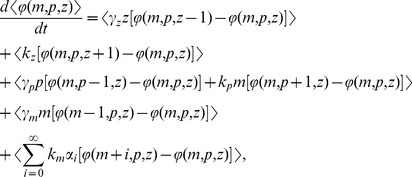
(23)where 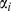 is given by (21). Equation (24) yields moment dynamics identical to (14) except for the time derivative of 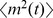. For 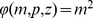,



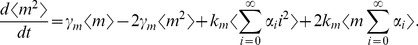
(23)Using the fact that for a geometric distribution
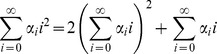
(24)and (21), (23) is written as



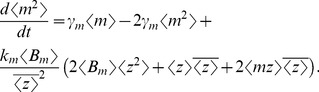
(25)Steady-state analysis of (14) (with (14c) replaced by (25)) results in
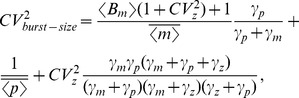
(26)the total protein noise level for transcriptional burst size fluctuations. As expected when 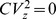 (no parameter fluctuations) (26) reduces to (10). Comparison of (26) with (15) reveals that for a given 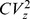, burst size fluctuations generates larger variability in protein level than burst frequency fluctuations.

### Computation of Intrinsic and Extrinsic Noise

For burst size fluctuations, the deterministic model used for quantifying extrinsic noise will be identical to (16). Since both transcriptional burst size and frequency appear together, replacing 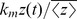 with 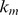, and 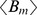 with 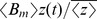 in (16) does not alter the model. Thus, extrinsic noise is same irrespective of whether fluctuations are in the transcriptional burst size or frequency. Using (19c) and (26)
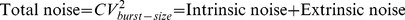
(27a)

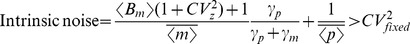
(27b)

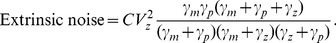
(27c)


**Figure 3:**
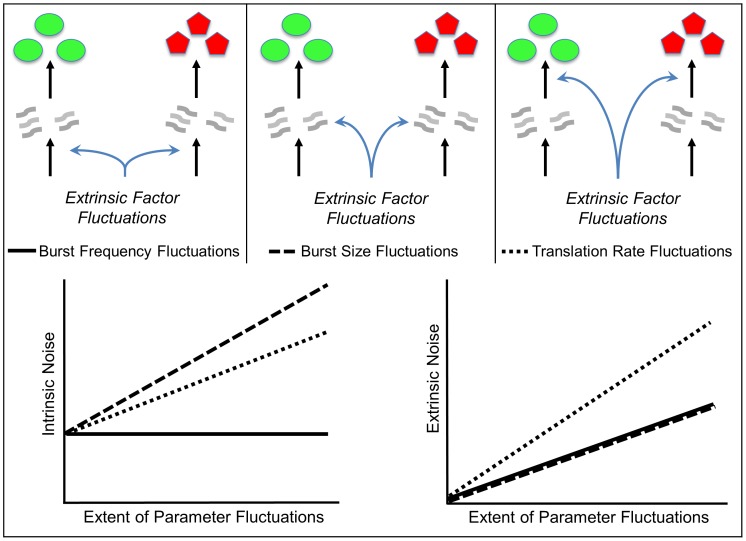
Gene expression variability for individual-parameter fluctuations. Intrinsic and extrinsic noise measured in two-color assay as a function of 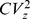 (extent of parameter fluctuations) for fluctuations in the transcription burst frequency (left), transcription burst size (middle) and mRNA translation rate (right). Intrinsic noise is independent of 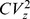 for transcription burst frequency fluctuations. However, for transcription burst size or translation rate fluctuations, intrinsic noise increases with 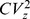. Extrinsic noise always increases with 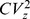 and is the largest for translation rate fluctuations.

In contrast to (19), intrinsic noise linearly increases with 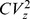 for burst size fluctuations (Figure 3).

## Translation Rate Fluctuations

Next, we consider mRNA translation rate fluctuations and set it equal to 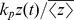. From Figure 2, this implies that the propensity function for the translational event is now nonlinear and given by 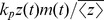. Since mRNA production is no longer dependent on 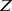, 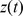 and 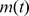 are independent random processes.

### Computation of Total Noise

Statistical moments of 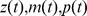 are obtained from (13) with 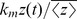 replaced by 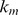, and 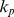 replaced by 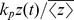. Using the fact that 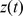 and 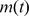 are independent yields
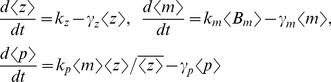
(28a)

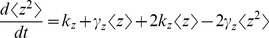
(28b)

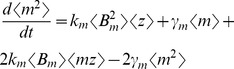
(28c)

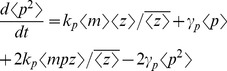
(28d)

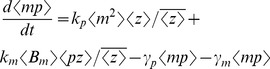
(28e)

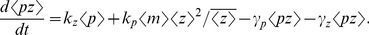
(28f)


Note that the moment dynamics is not closed, in the sense that, the time derivative of the second order moments 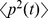 depends on the third order moment 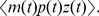 This phenomenon occurs due to nonlinear propensity functions and typically closure methods are needed to solve for the moments [56], [57]. The independence of 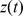 and 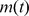 is exploited for moment closure. More specifically,
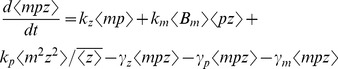
(29)which is dependent on the fourth order moment 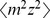. As
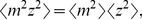
(30)equations (28)–(30) form a closed system of equations that yield total variability in protein level as
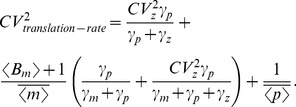
(31)


### Computation of Intrinsic and Extrinsic Noise

Strategy for decomposing (31) into its intrinsic/extrinsic components is similar to previous sections: extrinsic noise is first computed from a deterministic model and then subtracted from (31) for the intrinsic noise. Consider the differential equation model
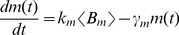
(32a)

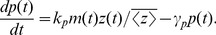
(32b)with translation rate fluctuations. Replacing 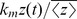 by 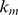, and 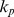 by 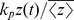 in (17), we obtain moment dynamics identical to (28) except for



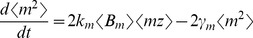
(33a)

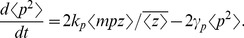
(33b)


Steady-state analysis of (28)–(30) (with (28c)–(28d) replaced by (33a)–(33b)) yields
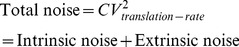
(34a)

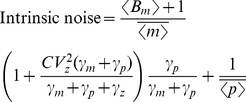
(34b)

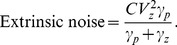
(34c)


As in (27), fluctuations in the translation rate enhance both intrinsic and extrinsic noise (Figure 3).

## Simultaneous Model Parameter Fluctuations

Previous sections focused on expression variability generated by fluctuations in individual parameters. However, stochasticity in the abundance of certain cellular factors (such as ATP) can simultaneously affect both transcription and translation. Motivated by this scenario, we investigate how perfectly correlated fluctuations in the transcription rate (measured by either the transcriptional burst frequency or burst size) and translation rate affect intrinsic and extrinsic noise.

### Transcription Burst Frequency and Translation Rate Fluctuations

Assume transcriptional bursts occur at a rate 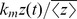 with a geometrically distributed burst size independent of 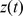 and given by (4). Each mRNA produces proteins at a rate 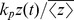, which is perfectly correlated with burst frequency. Let
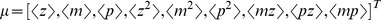
(35)be a vector containing all the first and second order moments of the population counts. Then, using (13) with 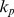 replaced by 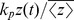, time evolution of 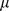 can be compactly represented as
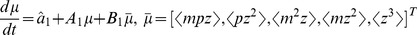
(36)where vector 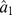, matrices 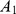, 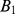 depend on model parameters and 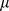 is a vector of third order moments. As one would expect, nonlinear propensity function for the translation event leads to unclosed moment dynamics. It turns out that incorporating certain higher order moments in 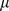 can close moment equations. More specifically, the time derivative of
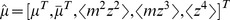
(37)is closed and is given by
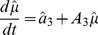
(38)for some vector 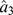 and matrix 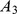. Steady-state analysis of (38) results in an exact analytical formula for the total steady-state protein noise level. In previous sections (individual parameter fluctuations), average protein copy number was invariant of 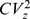 and given by (8). However, simultaneous transcription/translation rate fluctuations enhance mean protein level from (8) to



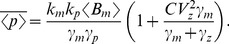
(39)To resolve total noise into its intrinsic/extrinsic components the following deterministic model is used
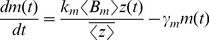
(40a)

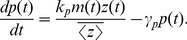
(40b)


**Figure 4:**
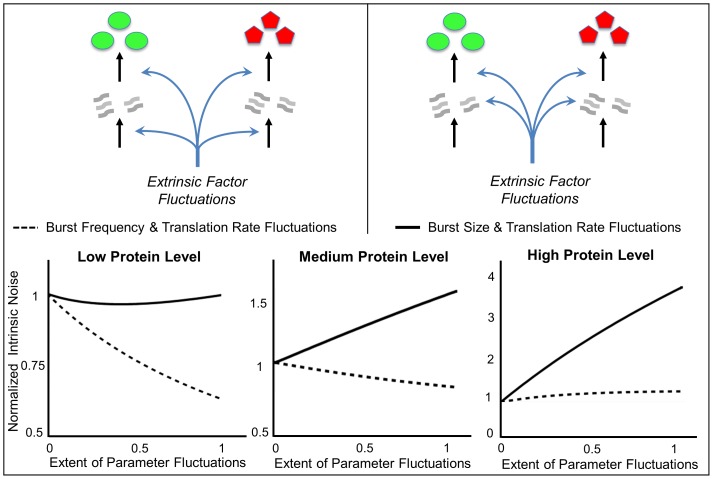
Gene expression variability for multiple-parameter fluctuations. Intrinsic noise measured in two-color assay as a function of 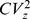 (extent of parameter fluctuations) for simultaneous fluctuations in the transcription burst frequency/translation rate (left), and transcription burst size/translation rate (right). The latter case generates larger intrinsic noise and also yields different qualitative trends compared to burst frequency/translation rate fluctuations. Depending on parameter regimes, intrinsic noise can increase, decreases or change non-monotonically with 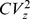. High, medium, low protein populations correspond to an average of 300, 30 and 10 protein copies per cell, respectively. Other model parameters taken as mRNA half-life = 2 hours, protein half-life = time-scale of parameter fluctuations = 10 hours, mean transcriptional burst size = 10 and mean mRNA copy number per cell = 50.

For (40), the moment generator equation is obtained by replacing 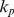 with 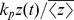 in (17). Performing an identical analysis as (36)–(38) for the hybrid model (40) yields the extrinsic noise, which is subtracted from the total noise to obtain the intrinsic noise. Unfortunately, these expressions are too complex to be listed here but are illustrated in Figure 4. Interestingly, simultaneous fluctuations in the burst frequency and translation rate can either increase or decrease intrinsic noise depending on model parameters.

To further elucidate the relationship between intrinsic noise and 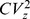, the case of slow fluctuations in 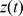 compared to mRNA/protein turnover rates (i.e., 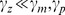) is considered. In this case noise expressions reduce to
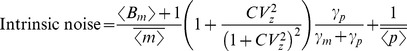
(41a)

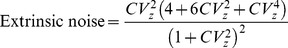
(41b)where the mean mRNA and protein levels are given by (see (39))



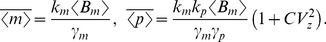
(42)Equation (41a) reveals that when
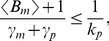
(43)intrinsic noise monotonically decreases with 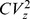. On the other hand when
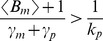
(44)intrinsic noise first increases with 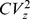, reaches a maximum at
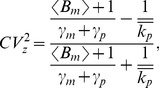
(45)and then decreases with increasing 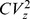.

### Transcription Burst Size and Translation Rate Fluctuations

Let transcriptional bursts occur at a constant rate 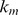 with a geometrically distributed burst size that is dependent on 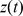 and given by (21). mRNA translation rate is assumed to be perfectly correlated with burst size and is set equal to 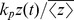. The time evolution of moments is obtained from (22) with 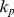 replaced by 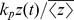. As in the previous section, although the time derivative of 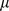 (Eq. (35)) is not closed, the evolution of 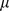 (Eq. (37)) is given by a closed system of linear equations that yield an exact expression for the total protein noise level. Recall that extrinsic noise is similar for transcription burst size and burst frequency fluctuations. Hence, calculation of extrinsic noise for model (40) is used to resolve the total noise into its intrinsic and extrinsic components. These results show that simultaneous transcription burst size/translation rate fluctuations not only generate a larger intrinsic noise but also have qualitatively different trends compared to burst frequency/translation rate fluctuations (Figure 4).

For slow fluctuations in 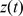 compared to mRNA/protein turnover rates
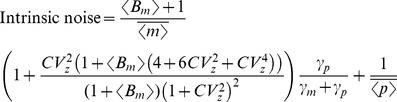
(46a)

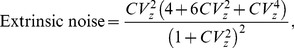
(46b)where 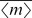 and 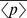 are given by (42). Analysis of (46a) shows that when
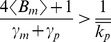
(47)intrinsic noise increases with 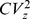. However, when
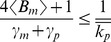
(48)intrinsic noise first decreases with increasing 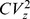 and then increases (Figure 4).

## Discussion

Given the different functional roles of gene expression noise inside cells [3], [32], much work has focused on understanding how variations in the level of a protein arises between otherwise identical cells. A class of models were introduced where stochasticity arises from two sources: i) Random production and degradation of individual mRNA transcripts/protein molecules stemming from the inherent probabilistic nature of biochemical reactions and ii) Fluctuations in model parameters that correspond to randomness in cell-specific factors. Exact analytical formulas for total variability in protein level were derived, in spite of the fact that in many cases parameter fluctuations lead to nonlinear propensity functions. These formulas were decomposed into intrinsic and extrinsic noise components as measured by the two-color reporter assay (Figure 1).

### Which Mechanism Generates the Largest Gene Expression Noise?

#### Individual-parameter fluctuations

Comparison of (19), (27) and (34) shows that for low values of 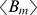, fluctuations in the translation rate create the most variability in protein copy numbers. On the other hand for high 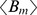, burst size fluctuations generate the most variability. Burst frequency fluctuations always generate the lowest noise.

#### Multiple-parameter fluctuations

Equations (41) and (46) reveal that simultaneous fluctuations in translation and transcription rates can dramatically increase expression variability. For example, consider protein half-life = time-scale of parameter fluctuations = 24 hours, mRNA half-life = 8 hours, mean mRNA count/cell = 100, 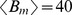 and 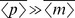. Then, for constant parameters, 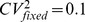 (Eq. (10)). Assuming ATP affects transcriptional burst size and translation rate, 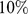 variability in ATP abundance (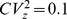) enhances noise level three-fold from 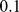 to 0.32. In comparison, burst size fluctuations of similar magnitude only increase 0.1 to 0.16. These results reinforce recent observations that intercellular variation in ATP abundance can be a major driver of gene expression noise [43]. An implicit assumption in this analysis is that protein and mRNA degradation is insensitive to ATP. Since both ATP-dependent and ATP-independent degradation pathways exist within cells, further work on ATP-sensitive degradation rates is clearly needed.

### Relationship between Intrinsic Noise and 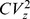


Using Monte Carlo simulation techniques previous studies had shown that parameter fluctuations can alter intrinsic noise measurements in a two-color assay [42], [60]. Building up on these results, a systematic analytical analysis of how fluctuations in both individual and multiple model parameters affect randomness in protein populations counts was performed. Main findings are as follows:

- Intrinsic noise is invariant of fluctuations in the transcription burst frequency (i.e., how often mRNA bursts occur from the promoter).
- Intrinsic noise increases with 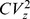 (extent of parameter fluctuations) for fluctuations in the transcription burst size (i.e., mean number of mRNAs produced in each burst) or mRNA translation rate.
- For simultaneous fluctuations in the burst frequency and translation rate, intrinsic noise decreases with 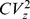 for low protein abundance (Figure 4). Intuitively, for low protein abundance (as determined by (43)), the Poissonian term 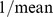 has a significant contribution to intrinsic noise (second term on the right-hand-side of (41a)). Simultaneous fluctuations increase mean protein level (see (39)), decreasing intrinsic noise. For high protein abundance, ignoring the second term in (41a) yields
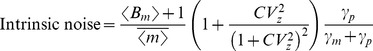
(49)which first increases, and then decreases with 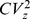. The maximal value is achieved at 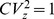.
- Simultaneous fluctuations in the transcription burst size and translation rate typically increases intrinsic noise. However, for low protein abundance intrinsic noise exhibits a U-shape profile with 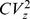 (Figure 4).
- In contrast to intrinsic noise, extrinsic noise always monotonically increases with 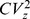.

We comment on how these trends change if Fano factor (variance/mean), instead of coefficient of variation, is used for quantifying noise. This is particularly important in the case of multiple-parameter fluctuations, where mean protein levels are dependent on 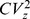 (see (39)). Our analysis shows that in contrast to the above trends, the intrinsic noise Fano factor always monotonically increases with 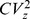 for simultaneous fluctuations in the transcription and translation rates.

Recall that our results correspond to a model where mRNAs are produced in instantaneous transcriptional bursts. For a promoter that stochastically toggles between active and inactive states, this approximation corresponds to an unstable active state [47], where the promoter quickly transitions back to the inactive state after producing a burst of mRNA transcripts form the active state. It turns out that some of the above intrinsic noise versus 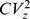 trends are also valid outside the instantaneous burst limit. For example, Monte Carlo simulations have shown that for fluctuations in the translation rate or transcription burst size, intrinsic noise increases with 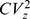 when promoter spends a finite amount of time in active and inactive states [60]. Future work will extend analytical formulas for intrinsic and extrinsic noise to cases where the promoter stochastically transitions between different transcriptional states.

### Estimation of Model Parameters from Noise Measurements

Gene expression noise is often used to calculate the mean transcriptional burst size and frequency for a specific gene or promoter [40], [48], [51], [52]. Recall from (10) that for fixed model parameters
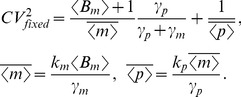
(50)


Given measurements of 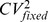 and 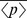, a priori knowledge of 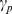, 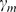, 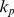, mean burst size 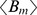 and frequency 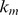 can be computed from (50). Typically, 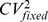 is assumed to be equal to the intrinsic noise measured in a two-color assay. However, our results show that this is only valid for transcription burst frequency fluctuations. For all other cases, 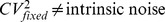, and using intrinsic noise for 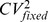 in (50) will lead to erroneous parameter estimates [42].

Analytical formulas developed here can be used to back calculate 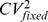 from intrinsic and extrinsic noise measurements. This point is illustrated for the physiologically relevant parameter regime
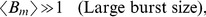
(51a)

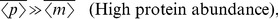
(51b)

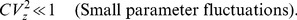
(51c)


In this regime, intrinsic noise is expressed as
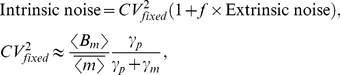
(52)where 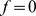 for burst frequency fluctuations and 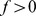 in all other cases. Analytical expressions for 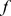 are provided in the Text S1, and it depends only on mRNA, protein turnover rates and time-scale of parameter fluctuations (more specifically on ratios 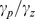 and 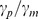). Consider a stable reporter protein where 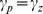 = time-scale of cell division, and 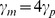, then



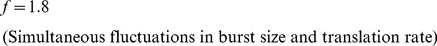
(53a)

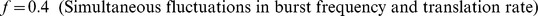


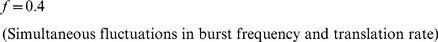
(53b)

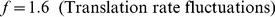
(53c)

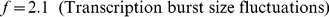
(53d)

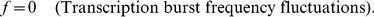
(53e)


Therefore, if 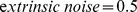 in an experiment, from (52) and (53d), 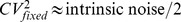 for burst size fluctuations. Traditional approach of assuming 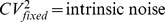 would overestimate 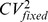 by 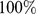. Using 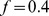 for simultaneous burst frequency/translation rate fluctuations gives 
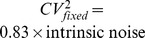
, and 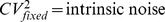 may not be a bad approximation in this case. It can be shown that
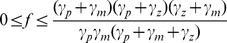
(54)with upper (lower) bound being realized for burst size (frequency) fluctuations. Without prior knowledge on the source of extrinsic noise, (54) yields the following bounds on 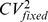:
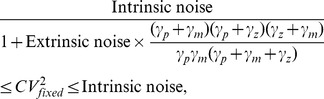
(55)for the physiologically relevant parameter regime (51). Thus, our results provide the necessary correction factors for accurately determining 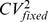 from two-color reporter experiments, which would be useful for estimating 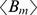 and 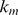.

In conclusion, our analysis reveals how stochastic synthesis and degradation of biomolecules combines with parameters fluctuations to generate heterogeneity in protein level across a clonal cell population. These results will help understand how stochastic variability is regulated inside cells, and for extracting meaningful information from single-cell gene expression measurements. Future work will consider scenarios where randomness in cellular factor levels simultaneously affects synthesis and degradation pathways, or only degradation. Unfortunately, exact solutions are unavailable in many of these cases. However, preliminary analysis has found moment closure techniques useful for obtaining closed-form solutions for the statistical moments. A recent study has generalized notions of intrinsic and extrinsic noise from statistical moments to temporal correlations [61]. In particular, the auto-correlation function of 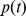 can be decomposed into intrinsic and extrinsic components based on the two-color assay [61]. Future will work will derive analytical expressions for protein auto-correlation and cross-correlation functions in stochastic models with parameter fluctuations, and study how noise signature within them can be used for probing genetic systems.

## Supporting Information

Text S1 **Formulas for factor** *f* **in Eq. 52.**
(PDF)
