# Supplementary material for: Quantifying Intrinsic and Extrinsic Variability in Stochastic Gene Expression Models
Source: PLoS One. 2013 Dec 31;8(12):e84301. doi: 10.1371/journal.pone.0084301 (PMC3877255; doi:10.1371/journal.pone.0084301)
Supplement: Text S1 — Formulas for factor f in Eq. 52. (PDF) [file pone.0084301.s001.pdf]

Formulas for factor f in Eq. 52

Simultaneous fluctuations in mRNA translation rate and transcription burst size

$$f = \frac{(\gamma_m + \gamma_p)(\gamma_p + \gamma_z)\left(8\gamma_m^2(\gamma_m + \gamma_p) + 2\gamma_m(7\gamma_m + 6\gamma_p)\gamma_z + (7\gamma_m + 2\gamma_p)\gamma_z^2 + \gamma_z^3\right)}{\gamma_p(2\gamma_m + \gamma_z)(\gamma_m + \gamma_p + \gamma_z)\left(4\gamma_m(\gamma_m + \gamma_p) + (4\gamma_m + \gamma_p)\gamma_z\right)}$$

Simultaneous fluctuations in mRNA translation rate and transcription burst freq.

$$f = \frac{(\gamma_m + \gamma_p)(\gamma_p + \gamma_z)\left(2\gamma_m^2(\gamma_m + \gamma_p) + \gamma_m(3\gamma_m + 5\gamma_p)\gamma_z + (\gamma_m + \gamma_p)\gamma_z^2\right)}{\gamma_p(2\gamma_m + \gamma_z)(\gamma_m + \gamma_p + \gamma_z)\left(4\gamma_m(\gamma_m + \gamma_p) + (4\gamma_m + \gamma_p)\gamma_z\right)}$$

---

mRNA translation rate fluctuations

$$f = \frac{(\gamma_m + \gamma_p)(\gamma_p + \gamma_z)}{\gamma_p(\gamma_m + \gamma_p + \gamma_z)}$$

Transcription burst size fluctuations

$$f = \frac{(\gamma_m + \gamma_p)(\gamma_m + \gamma_z)(\gamma_p + \gamma_z)}{\gamma_m\gamma_p(\gamma_m + \gamma_p + \gamma_z)}$$

Transcription burst frequency fluctuations

$$f = 0$$
